# Prevalence of cancer therapy cardiotoxicity as assessed by imaging procedures: A scoping review

**DOI:** 10.1002/cam4.5854

**Published:** 2023-03-31

**Authors:** Valeria Cantoni, Roberta Green, Roberta Assante, Adriana D'Antonio, Francesca Maio, Emanuele Criscuolo, Roberto Bologna, Mario Petretta, Alberto Cuocolo, Wanda Acampa

**Affiliations:** ^1^ Department of Advanced Biomedical Sciences University of Naples Federico II Naples Italy; ^2^ IRCCS SYNLAB SDN Naples Italy

**Keywords:** cancer therapy, cardiotoxicity, imaging

## Abstract

**Background:**

Advances in treatment and optimization of chemotherapy protocols have greatly improved survival in cancer patients. Unfortunately, treatment can cause a reduction in left ventricular (LV) ejection fraction (EF) leading to cancer therapy‐related cardiac dysfunction (CTRCD). We conducted a scoping review of published literature in order to identify and summarize the reported prevalence of cardiotoxicity evaluated by noninvasive imaging procedures in a wide‐ranging of patients referred to cancer treatment as chemotherapy and/or radiation therapy.

**Methods:**

Different databases were checked (PubMed, Embase, and Web of Science) to identify studies published from January 2000 to June 2021. Articles were included if they reported data on LVEF evaluation in oncological patients treated with chemotherapeutic agents and/or radiotherapy, measured by echocardiography and/or nuclear or cardiac magnetic resonance imaging test, providing criteria of CTRCD evaluation such as the specific threshold for LVEF decrease.

**Results:**

From 963 citations identified, 46 articles, comprising 6841 patients, met the criteria for the inclusion in the scoping review. The summary prevalence of CTRCD as assessed by imaging procedures in the studies reviewed was 17% (95% confidence interval, 14–20).

**Conclusions:**

The results of our scoping review endorse the recommendations regarding imaging modalities to ensure identification of cardiotoxicity in patients undergoing cancer therapies. However, to improve patient management, more homogeneous CTRCD evaluation studies are required, reporting a detailed clinical assessment of the patient before, during and after treatment.

## 1 INTRODUCTION

Lately, early diagnosis, progress in cancer treatment and optimization of chemotherapy protocols have improved survival in cancer patients in a meaningful way. Nevertheless, conventional and oncologic therapies have a broad range of adverse cardiac events, including myocardial toxicity.^1^ Cardio‐oncology is a relatively new area of interest focusing on the identification, monitoring, and treatment of cardiovascular disease that occurs as a side effect of cancer treatments.^2^ Heart failure (HF) and ventricular dysfunction represent the most troubling adverse effects. The prevalence of subclinical left ventricular (LV) dysfunction may be found as far as 42% of cancer patients in recruited treatment groups.^3^ HF and LV dysfunction due to therapy for cancer are associated with a 3.5‐fold increase in the mortality risk.^4^ However, the frequency of cardiotoxicity depends on several variables related to cancer treatment and to patient characteristics.^5^

Cancer therapy‐related cardiac dysfunction (CTRCD) has been commonly defined as a reduction in LV ejection fraction (EF) ≥10% to a value of <50% or as a reduction in LVEF below 53% or an absolute decrease in LVEF >20%.^6^, ^7^, ^8^ However, the categorization of the severity of HF and LV dysfunction as markers of cancer therapy cardiotoxicity is extensively heterogeneous.^9^ Endomyocardial biopsy is the gold standard for the diagnosis of cardiomyocyte damage, but this procedure is hardly used due to the invasiveness and low availability.^10^ Noninvasive diagnostic imaging techniques as echocardiography, cardiac magnetic resonance (CMR), and nuclear testing have been widely used for the evaluation of CTRCD.^11^

Several studies focused on the role of noninvasive diagnostic imaging techniques such as echocardiography, CMR, and nuclear cardiology in the evaluation of CTRCD.^11^ The large volume and the heterogeneity of published studies, related to type of cancer patients, clinical characteristics of patients, treatment adopted, CRTD definition, and the method used for the diagnosis of CRTD highlight a relevant need to organize and summarize findings so that the most current and accurate information can be easily accessed. In this scenario, we conducted a scoping review of published literature designed in order to identify and summarize the available data on prevalence of cardiotoxicity evaluated by noninvasive imaging procedures in a wide‐ranging of oncological patients treated with chemotherapy or radiation therapy, in order to give an updated picture of what is known about.

## 2 MATERIALS AND METHODS

We performed a review of the medical literature using the standard methodology for scoping literature review as published by the Cochrane Collaboration, and according to the Preferred Reporting Items for Systematic Reviews and Meta‐Analyses (PRISMA) statement (see the Appendix S1 for PRISMA Checklist).^12^

PubMed, Embase, and Web of Science databases were screened to identify studies published from January 2000 to June 2021. Articles search was limited to data retrieved in humans and adults and was performed adopting the following keywords: “cardio‐oncology, cardiotoxicity, chemotherapy, radiotherapy, cardio‐imaging, left ventricular ejection fraction, echocardiography, ultrasound, cardiac magnetic resonance (OR CMR), nuclear imaging.” The complete search strategy is depicted in the Appendix S2. A screening for appropriateness of the title and abstract of potentially pertinent articles was conducted by two reviewers (V.C. and R.G.) before retrieval of the full article, and disagreements were resolved by consensus. The full‐published studies of the abstracts identified by the reviewers were downloaded, and they individually conducted the final selection relying on the eligibility criteria; disagreements were solved by consensus. Moreover, the bibliographies of retrieved studies were manually screened for further citations.

Each article was identified evaluating journal, authors, and year of publication. To harmonize the predictors of interest, a publication was considered eligible if all of the following criteria were met: (1) the study reported LVEF data in patients with cancers treated with chemotherapeutic agents and/or radiotherapy; (2) the study provided LVEF data by echocardiography and/or nuclear test and/or CMR evaluated before and after chemotherapeutic agents and/or radiotherapy; (3) the study provided criteria of CTRCD evaluation such as the specific threshold for the LVEF decrease; and (4) follow‐up was at least 3 months after therapy completion. Articles were included if data were obtained from retrospective, prospective, or observational studies. In case of different studies from the same research team, potential patient population duplication was prevented by including the largest cohort only.

Patient population data were retrieved on age and on prevalence of female gender, cancer type, anticancer therapies, cardiac assessment modality, follow‐up time, and cardiovascular risk factors such as diabetes, hypertension, dyslipidemia, smoking, family history of coronary artery disease (CAD), and history of CAD (including previous myocardial infarction and coronary revascularization). All articles were evaluated for methodological quality by the use of Joanna Briggs Institute Prevalence Critical Appraisal Tool.^13^

The criteria observe the following issues: representative sample ensured, appropriate recruitment ensured, adequate sample size, appropriate description and reporting of study subjects and setting, data of the identified sample adequate, the condition was measured reliably and objectively, appropriate statistical analysis, confounding factors, subgroups, differences identified and accounted for. There are four possible responses for these questions: yes, no, unclear, or not applicable.^13^ Two reviewers (V.C. and R.G.) assessed the risk of bias in each eligible article individually. Disagreements were solved by consensus. If the answers to all the signal problems were “yes,” a low risk of bias was attributed to the study; if the answers to all the signal problems had one or more “no” or “unclear” values, an unclear risk of bias was used; if the answers to all the signal problems contained at least one “no” but no “yes” answers, a high risk of bias was attributed.

Given the disparity of study designs, treatment, and population in the literature considered, a descriptive summary approach was used with the results presented in narrative form and in tables. However, a quantitative synthesis was also performed to calculate a summary estimate of the prevalence of cardiotoxicity. A quantitative synthesis was also performed to calculate a summary estimate of the prevalence of cardiotoxicity in overall population and according to the year of publication (between 2000–2010 and 2011–2021). The logit transformation was used to pool individual studies proportions and to present in a forest plot weighted estimates with inverse‐variance weights obtained from a random‐effects model; study specific 95% confidence intervals (CI) were calculated using the exact method.^14^ The I‐squared statistic was used to assess the heterogeneity of included studies.^15^

## 3 RESULTS

**FIGURE 1:**
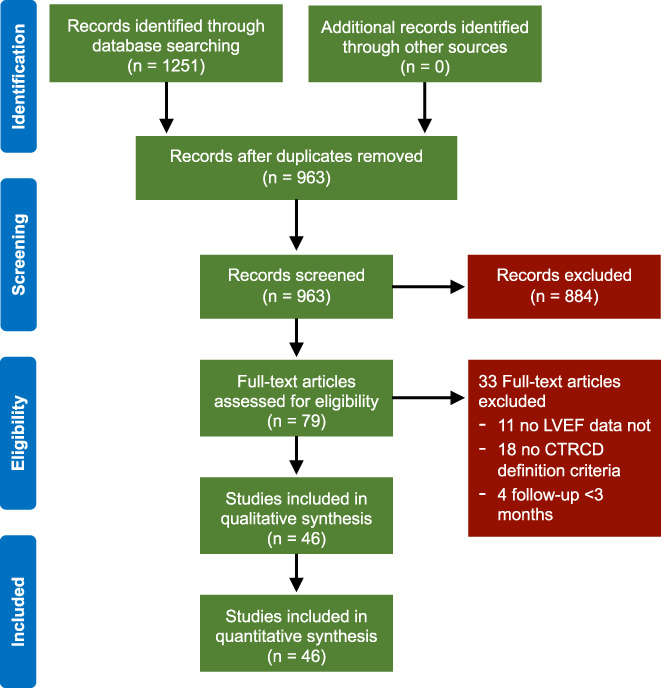
Study selection process.

The PRISMA flowchart is depicted in Figure 1. The databases search identified 1251 potentially eligible records. Among these, 288 were duplicates and then discharged, leaving 963 citations. The reviewers removed 884 citations evaluating the appropriateness of titles and abstracts of these studies, leaving 79 articles. Then, each reviewer blindly evaluated the full text of these articles, excluding 33 articles. Finally, 46 articles including 6841 patients were analyzed.

**FIGURE 2:**
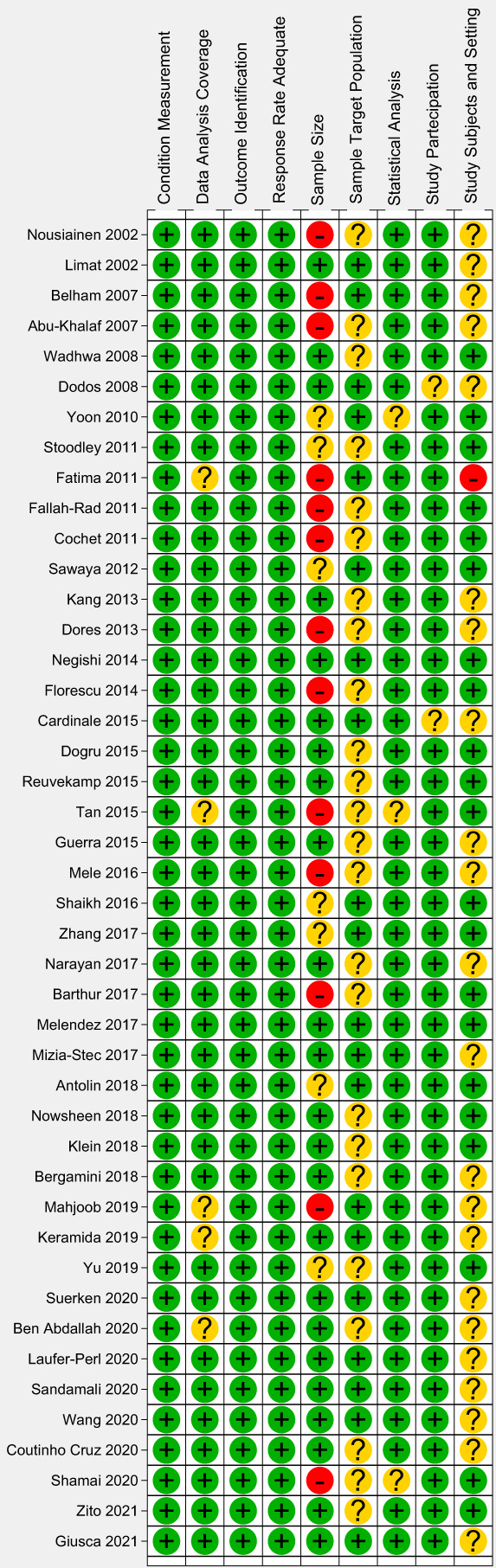
Methodological quality of the included studies assessed with Joanna Briggs Institute Critical Appraisal tool for risk of bias and applicability concerns. The green circle represents low risk of bias, the yellow circle unclear risk of bias, and the red circle high risk of bias.

The quality assessment of included were summarized in Figure 2. The domains that showed an unclear risk of bias were “study subjects and setting” and “sample target population.” The domain that showed a high risk of bias was “sample size.” These results could be due to the lack of description of patient characteristics and small number of patients evaluated in some studies.

Table 1 showed the demographic data and clinical characteristics of patients.^16^, ^17^, ^18^, ^19^, ^20^, ^21^, ^22^, ^23^, ^24^, ^25^, ^26^, ^27^, ^28^, ^29^, ^30^, ^31^, ^32^, ^33^, ^34^, ^35^, ^36^, ^37^, ^38^, ^39^, ^40^, ^41^, ^42^, ^43^, ^44^, ^45^, ^46^, ^47^, ^48^, ^49^, ^50^, ^51^, ^52^, ^53^, ^54^, ^55^, ^56^, ^57^, ^58^, ^59^ Cancer type, treatment, and imaging technique for each study are reported in Table 2.^16^, ^17^, ^18^, ^19^, ^20^, ^21^, ^22^, ^23^, ^24^, ^25^, ^26^, ^27^, ^28^, ^29^, ^30^, ^31^, ^32^, ^33^, ^34^, ^35^, ^36^, ^37^, ^38^, ^39^, ^40^, ^41^, ^42^, ^43^, ^44^, ^45^, ^46^, ^47^, ^48^, ^49^, ^50^, ^51^, ^52^, ^53^, ^54^, ^55^, ^56^, ^57^, ^58^, ^59^ Patient population ranged from 28 to 2625 subjects. Mean age ranged from 44 to 62 years, with the prevalence of women ranging from 29% to 100%. Mean follow‐up was 9.7 ± 1.3 months.

**TABLE 1:** Demographic data and clinical characteristics of patients.

	Patients (*n*)	Female (%)	Age (year)	Hypertension (%)	Dyslipidemia (%)	Smoking (%)	Family history of CAD (%)	Diabetes (%)	Prior CAD (%)
Nousiainen et al.^16^	28	39	53	14	‐	‐	‐	‐	3
Limat et al.^17^	135	43	59	‐	‐	‐	‐	‐	‐
Belham et al.^18^	51	29	50 ± 18	10	‐	‐	‐	2	3
Abu‐Khalaf et al.^19^	32	100	57	‐	‐	‐	‐	‐	‐
Wadhwa et al.^20^	152	100	52 ± 10	11	13	17	20	7	2
Dodos et al.^21^	100	52	46 ± 1	18	‐	‐	‐	‐	‐
Yoon et al.^22^	88	53	52	32	26	‐	‐	17	7
Stoodley et al.^23^	52	100	49 ± 9	25	21	25	‐	4	6
Fatima et al.^24^	42	74	44 ± 10	0	‐	‐	‐	0	‐
Fallah‐Rad et al.^25^	42	100	47 ± 9	12	36	17	29	14	‐
Cochet et al.^26^	118	100	58	9	‐	13.5	‐	7	‐
Sawaya et al.^27^	81	100	50 ± 10	32	22	7	‐	1	‐
Kang et al.^28^	75	59	53 ± 13	13	‐	29	‐	4	‐
Dores et al.^29^	51	100	55 ± 14	35	25	5	19	11	‐
Negishi et al.^30^	159	80	49 ± 14	20	18	38	‐	6	‐
Florescu et al.^31^	40	100	51 ± 8	0	12	30	‐	0	‐
Cardinale et al.^32^	2625	74	50 ± 13	23	7.5	18	7	4	3
Dogru et al.^33^	50	46	45 ± 13	53	20	26	‐	6	‐
Reuvekamp et al.^34^	77	100	53 ± 9	14	‐	16	‐	4	‐
Tan et al.^35^	29	100	50 ± 10	24	21	10	‐	0	‐
Guerra et al.^36^	69	96	56 ± 13	42	19	16	29	6	‐
Mele et al.^37^	30	99	53 ± 11	30	30	20	33	7	‐
Shaikh et al.^38^	80	45	62 ± 14	52	43	‐	‐	26	16
Zhang et al.^39^	82	50	50 ± 12	‐	‐	‐	‐	‐	‐
Narayan et al.^40^	135	100	48	27	17	6	‐	8	‐
Barthur et al.^41^	41	100	52 ± 11	24	7	24	‐	10	2
Meléndez et al.^42^	112	70	52 ± 14	41	29	28	‐	17	1
Mizia‐Stec et al.^43^	67	46	58	42	‐	44.8	43	13	10
Antolín et al.^44^	142	100	49	21	17	‐	‐	‐	‐
Nowsheen et al.^45^	428	100	53 ± 12	33	18	16	‐	11	14
Klein et al.^46^	146	98	61 ± 12	29	8	6	2	9	1
Bergamini et al.^47^	162	100	59 ± 12	35	15	‐	‐	4	‐
Mahjoob et al.^48^	52	78	44	‐	‐	14	‐	‐	‐
Keramida et al.^49^	101	100	54 ± 11	15	‐	‐	‐	‐	1
Yu et al.^50^	47	100	52	17	11	30	‐	9	0
Suerken et al.^51^	71	68	54 ± 4	50	11	12	‐	17	4
Ben Abdallah et al.^52^	66	100	47 ± 9	‐	‐	‐	‐	‐	‐
Laufer‐Perl et al.^53^	237	70	62	38	23	31	‐	22	12
Sandamali et al.^54^	196	100	54 ± 11	0	‐	‐	‐	‐	0
Wang et al.^55^	65	52	51 ± 13	32	‐	31	‐	15	‐
Coutinho Cruz et al.^56^	105	100	54 ± 12	0	‐	‐	0	0	‐
Shamai et al.^57^	43	60.5	58 ± 16	37	19	26	‐	15	2
Zito et al.^58^	146	98	56 ± 11	35	25	20	‐	16	‐
Giusca et al.^59^	61	82	54 ± 15	36	15	7	‐	7	‐

Abbreviation: CAD, coronary artery disease.

**TABLE 2:** Cancer type, treatment, and imaging technique for each study.

	Cancer type	Treatment	Imaging	Definition of CTRCD
Nousiainen et al.^16^	LNH	ANT + RT	Nuclear	Decrease of LVEF >10% to ≤50%
Limat et al.^17^	LNH	ANT + RT	Nuclear	Decrease of LVEF ≥15% or decrease of LVEF to <50%
Belham et al.^18^	Different	ANT	Echo	Decrease of LVEF >10%
Abu‐Khalaf et al.^19^	Breast	ANT + TAX + RT	Nuclear	LVEF ≤50%
Wadhwa et al.^20^	Breast	TZB + RT	Nuclear	Decrease of LVEF ≥10% to <55% or decrease of LVEF ≥5% to <55% with signs or symptoms of HF
Dodos et al.^21^	Different	ANT	Echo	Decrease of LVEF >20% or decrease of LVEF >10% to <55% or HF
Yoon et al.^22^	Different	ANT + TZB	Echo‐Nuclear	LVEF <55%
Stoodley et al.^23^	Breast	ANT	Echo	Decrease of LVEF ≥10% to <50%
Fatima et al.^24^	Different	ANT	Echo‐Nuclear	Decrease of LVEF ≥10% to <50%
Fallah‐Rad et al. 2011^25^	Breast	ANT + TZB + RT	CMR	Decrease of LVEF >10% to <55% with signs or symptoms of HF
Cochet et al. 2011^26^	Breast	ANT + 5FU + TAX + TZB + RT	Nuclear	Decrease of LVEF ≥10% but <20% of baseline Decrease of LVEF <50% or ≥20% of baseline or HF
Sawaya et al.^27^	Breast	ANT + TZB + RT + TAX	Echo	Decrease of LVEF ≥10% to <55% or decrease of LVEF ≥5% to <55% with signs or symptoms of HF
Kang et al.^28^	LNH	ANT (CHOP)	Echo	Decrease of LVEF ≥10% to <55% or decrease of LVEF ≥5% to <55% with signs or symptoms of HF
Dores et al.^29^	Breast	ANT + TAX + TZB	Echo	LVEF <55% or decrease of LVEF >10%
Negishi et al.^30^	Different	ANT + TZB + RT	Echo	Decrease of LVEF >10% to <55%
Florescu et al.^31^	Breast	ANT	Echo	Decrease of LVEF ≥10% to <55% without signs or symptoms
Cardinale et al.^32^	Different	ANT + RT	Echo	Decrease of LVEF >10% to <50%
Dogru et al.^33^	LYM; Breast	ANT	Echo	LVEF <55%
Reuvekamp et al.^34^	Breast	ANT + RT + TZB	Nuclear	LVEF <50% or a drop of ≥10%
Tan et al.^35^	Breast	ANT + RT + TZB + TAX	Echo	Decrease of LVEF ≥10% to <55% or decrease of LVEF ≥5% to <55% with signs or symptoms of HF
Guerra et al.^36^	Breast	ANT + TAX	Echo	Decrease of LVEF ≥10% to <55% or decrease of LVEF ≥5% to <55% with signs or symptoms of HF
Mele et al.^37^	Breast	ANT + TAX + TZB + RT	Echo	Decrease of LVEF ≥10% to <55% or decrease of LVEF ≥5% to <55% with signs or symptoms of HF
Shaikh et al.^38^	AML	MITOXANTRONE	Echo	Decrease of LVEF ≥10% to <55% or decrease of LVEF ≥5% to <55% with signs or symptoms of HF
Zhang et al.^39^	LNH	ANT	Nuclear	Decrease of LVEF ≥10% to <50%
Narayan et al.^40^	Breast	ANT + TZB + RT	Echo	Decrease of LVEF ≥10% to <50%
Barthur et al.^41^	Breast	ANT + TZB + TAX + RT	CMR	Decrease of LVEF ≥10% to <55% or decrease of LVEF ≥5% to <55% with signs or symptoms of HF
Meléndez et al.^42^	Different	ANT + TAX + TZB + ALK	CMR	Decrease of LVEF >10% to <50%
Mizia‐Stec et al.^43^	LNH	ANT (CHOP) + RT	Echo	Decrease of LVEF ≥10%
Antolín et al.^44^	Breast	ANT + RT	Echo	LVEF <50%
Nowsheen et al.^45^	Breast	ANT + TZB	Echo	Decrease of LVEF ≥10% to <53%
Klein et al.^46^	Breast	ANT + TZB + RT	Nuclear	Decrease of LVEF <50% or decrease of LVEF >10%
Bergamini et al.^47^	Breast	ANT + TZB	Echo	Decrease of LVEF <50% or decrease of LVEF >10% with or without symptoms
Mahjoob et al.^48^	Different	ANT	Echo	Decrease of LVEF >10% to <53%
Keramida et al.^49^	Breast	TZB + RT	Echo	Decrease of LVEF ≥10% to <50%
Yu et al.^50^	Breast	RT + CHT	Echo	Decrease of LVEF ≥10% to <53% or decrease of LVEF >16%
Suerken et al.^51^	Different	ANT + TAX + TZB + CYCP	CMR	Decrease of LVEF ≥5% or a drop <50% or decrease of LVEF >10% to <53%
Ben Abdallah et al.^52^	Breast	ANT + 5 FU + RT + TAX	Echo	Decrease of LVEF >10% to <53%
Laufer‐Perl et al.^53^	Different	CHT + RT + TZB	Echo	Decrease of LVEF >10% to <53%
Sandamali et al.^54^	Breast	ANT + RT	Echo	Decrease of LVEF >10%
Wang et al.^55^	LNH	ANT	Echo	Decrease of LVEF >10% to <53%
Coutinho Cruz et al.^56^	Breast	ANT + RT	Echo	Decrease of LVEF >10% to <54%
Shamai et al.^57^	Sarcoma	ANT	Echo	Decrease of LVEF >10% to <53%
Zito et al.^58^	Breast	ANT	Echo	Decrease of LVEF ≥10% to <50%
Giusca et al.^59^	Different	ANT + TZB + RT + TAX + CYCP	CMR	Decrease of LVEF >10% to <53%

Abbreviations: ALK, alkylating agents; ANT, anthracycline; CHOP, cyclophosphamide, doxorubicin, oncovin and prednisone; CHT, different type of treatment; CMR, cardiac magnetic resonance; CTRCD, cancer therapeutics related cardiac dysfunction; CYCP, cyclophosphamide; Echo, echocardiography; FU, fluorouracil; HF, heart failure; LNH, lymphoma non Hodgkin; LVEF, left ventricular ejection fraction; LYP, lymphoma; RT, radiotherapy; TAX, taxane; TZB, trastuzumab.

**FIGURE 3:**
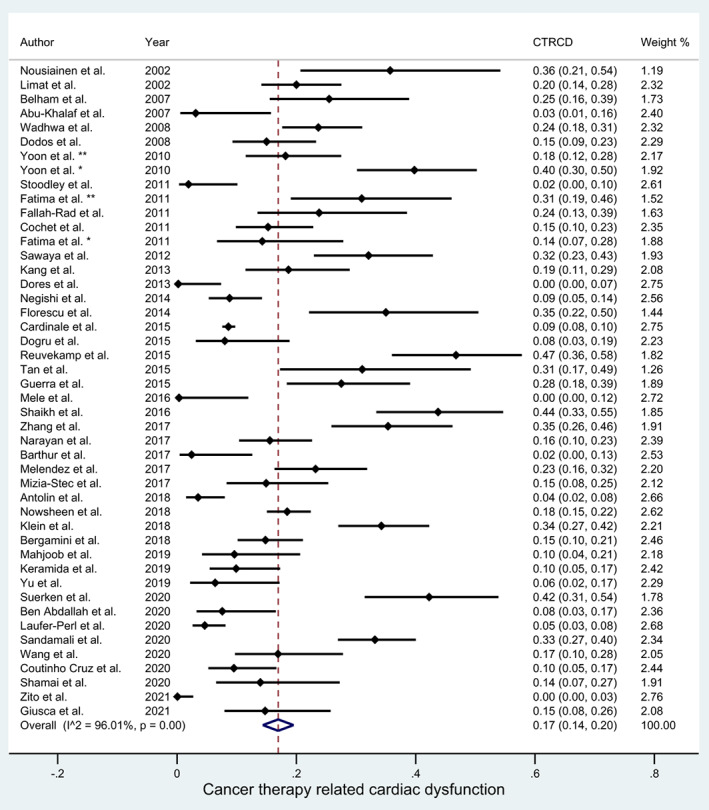
Forest plot of cancer therapeutics related cardiac dysfunction (CTRCD) prevalence in the overall studies. Horizontal lines represent 95% confidence interval (CI) of the point estimates. The diamond represents the pooled estimate (size of the diamond = 95% CI). The dashed vertical line represents the overall point estimate.

The summary prevalence of CTRCD assessed by imaging procedures in the studies reviewed was 17% (95% CI, 14–20) and the heterogeneity was 96% (Figure 3). The prevalence of CTRCD for studies published from 2011 to 2021 (16%; 95% CI, 13–19) was lower (*p* < 0.05) compared with studies published from 2000 to 2010 (22%; 95% CI, 14–29).

## 4 DISCUSSION

In our scoping review, we aimed to identify the incidence of cardiotoxicity in oncological patients by noninvasive imaging procedures in order to support clinicians in assessment and management of cardiotoxicity in oncological patients. As shown in Figure 3, the summary prevalence of cardiotoxicity in the studied population is around 17%.

Diagnosis of cardiac functional impairment plays a key role for clinical decision‐making in oncological patients referred to chemotherapy and/or radiation therapy. Moreover, a challenge for the diagnostic procedures should be the early assessment of cardiotoxicity. The Imaging and Cardio‐Oncology Study Groups of the HF Association analyzed the timely evidence for the role of cardiovascular imaging, such as echocardiography, CMR, CT, and nuclear testing, before and after cancer treatment.^11^ In addition, The International Cardio‐Oncology Society has recently developed criteria in the identification of CTRCD based on LVEF, echocardiographic global longitudinal strain, and blood biomarkers.^9^ In these documents, it was outlined that echocardiography is the first‐step imaging technique for the identification of cardiotoxicity through the evaluation of LVEF.^10^ Other echocardiographic indices, such as the global longitudinal LV strain, have been more recently introduced for the early identification of cardiac toxicity.^24^ Those indications have been confirmed and detailed by the recently published ESC guidelines, which have reported a clear scheduled timing follow‐up by prechemotherapy CAD patients' risk assessment and type of administrated chemotherapy showing the 3D echocardiography as the gold standard, using CRM and radionuclide angiography only when echocardiography is not available or not diagnostic.^60^

Recent evidence about the need of early diagnosis and rigorous follow‐up in cancer patients who underwent chemotherapy or radiotherapy led an incrementing effort in the definition of new protocols, within each diagnostic method, providing a timely diagnosis and a better patient management to cardiologists and oncologists.^61^, ^62^

Scoping reviews are a type of systematic review, focusing on large and heterogeneous body of literature relative to a research topic of interest. They are particularly useful for knowledge synthesis in case of lack of understanding of key conceptions within a topic and when a research topic is of a complex nature. In the field of cardio‐oncology, the large volume and the disparateness of published work, related to type of cancer patients, clinical characteristics of patients, the treatment adopted, CRTD definition, and the method used for the diagnosis of CRTD highlight a relevant need to organize and summarize findings so that the most current and accurate information can be easily accessed.

Our review indicated as a main issue the overall low quality of the included studies (Figure 2), mostly related to patient sample size and study design. Most of the included and analyzed studies enrolled a small number of patients. Moreover, most of them considered a prevalent female population undergoing chemotherapy for breast cancer, limiting the external validity for patients with other type of cancer and for male patients. Additionally, the patients' cohorts are characterized by heterogeneous cancer type and different chemotherapy protocol with different treatment duration time.

From our study, it also emerged that the summary prevalence of CTRCD was slightly lower for studies published from 2011 to 2020 as compared to those published from 2000 to 2010 (16% vs. 22%). It should be considered that the chemotherapies have significantly changed over time, especially those for breast cancer. Indeed, the large majority of studies evaluating CTRCD in breast cancer included in our search were published after 2010 where the therapy regiment reached an optimization in terms of pharmaceutical type, doses, cycles, and combined therapy. Furthermore, the improvement in regime treatments as well the evolution in the methods linked to each imaging procedure could have had a significant role in the reduction of CTRCD prevalence observed after 2010.^63^, ^64^

Taking into account the above‐quoted guidelines, more homogeneous CTRCD evaluation studies should be designed in the future, reporting a detailed clinical assessment of the patient before, during, and after treatment. Moreover, standardized imaging modality and follow‐up for each chemotherapy scheme are imperative to obtain homogenous data for a useful analysis. Limitations of our study may include the searching MEDLINE, which could not include all the studies published in the literature, even if we have chosen the most various patter of keywords on the topic. We decided to exclude from our MEDLINE search studies published before the 2000, to reach as much as possible the most recent clinical and imaging overview in the CTRCD evaluation. This literature analyses may be used as a starting point for future studies, which aim to analyze CTRCD in oncologic patients, understanding which kind of clinical and methodological errors should be avoided to reach a strong conclusion that may lead the ordinary clinical practice.

## 5 CONCLUSIONS

The findings of this scoping review endorse the recommendations regarding imaging modalities to ensure identification of cardiotoxicity in patients undergoing cancer therapies. However, to improve patient management, more homogeneous CTRCD evaluation studies are required, reporting a detailed clinical assessment of the patient before, during, and after treatment.

## AUTHOR CONTRIBUTIONS

**Valeria Cantoni:** Data curation (equal); formal analysis (equal); investigation (equal); methodology (equal); software (equal); writing – original draft (equal). **Roberta Green:** Data curation (equal); formal analysis (equal); investigation (equal); methodology (equal); software (equal); writing – original draft (equal). **Roberta Assante:** Data curation (equal); investigation (equal); methodology (equal); writing – original draft (equal); writing – review and editing (equal). **Adriana D'Antonio:** Data curation (equal); investigation (equal); writing – original draft (equal). **Francesca Maio:** Data curation (equal); investigation (equal); methodology (equal); writing – original draft (equal); writing – review and editing (equal). **Emanuele Criscuolo:** Data curation (equal); methodology (equal). **Roberto Bologna:** Data curation (equal); methodology (equal). **Mario Petretta:** Data curation (lead); formal analysis (lead); methodology (lead); software (lead); supervision (lead); writing – original draft (lead); writing – review and editing (lead). **Alberto Cuocolo:** Data curation (lead); methodology (lead); supervision (lead); writing – original draft (lead); writing – review and editing (lead). **Wanda Acampa:** Conceptualization (lead); data curation (lead); formal analysis (lead); investigation (lead); methodology (lead); supervision (lead); writing – original draft (lead); writing – review and editing (lead).

## CONFLICT OF INTEREST STATEMENT

The authors made no disclosures.

## Supporting information


Appendix S1.

Appendix S2.

## Data Availability

Not applicable.
