# Supplementary material for: Prevalence of cancer therapy cardiotoxicity as assessed by imaging procedures: A scoping review
Source: Cancer Med. 2023 Mar 31;12(10):11396–407. doi: 10.1002/cam4.5854 (PMC10242861; doi:10.1002/cam4.5854)
Supplement: Supplementary file 2 — Appendix S2. [file CAM4-12-11396-s001.docx]

**Supplementary Material**

**Detailed search strategy**

(“cardio-oncology” [All fields] AND “cardiotoxicity” OR “cardiotox” [All fields] AND (“chemotherapy” OR “anti-cancer therapies” [MeSH terms]) AND “radiotherapy” [All fields] AND “cardio-imaging” [All fields] AND “left ventricular ejection fraction” OR “LVEF” [MeSH terms] OR “left ventricular dysfunction” [All fields] AND “echocardiography” OR “ultrasound” [MeSH terms] AND “nuclear imaging” [All fields] OR “multigated acquisition scan” OR “MUGA”[MeSH terms] OR “equilibrium radionuclide angiogram” [All fields] OR “blood pool scan” [All fields] AND “cardiac magnetic resonance” OR “CMR” [All fields] OR “magnetic resonance imaging” OR “MRI” [MeSH terms])
